# Supplementary material for: Glycosylceramide modifies the flavor and metabolic characteristics of sake yeast
Source: PeerJ. 2018 May 10;6:e4768. doi: 10.7717/peerj.4768 (PMC5949206; doi:10.7717/peerj.4768)
Supplement: Supplemental Information 1 [file peerj-06-4768-s001.pdf]

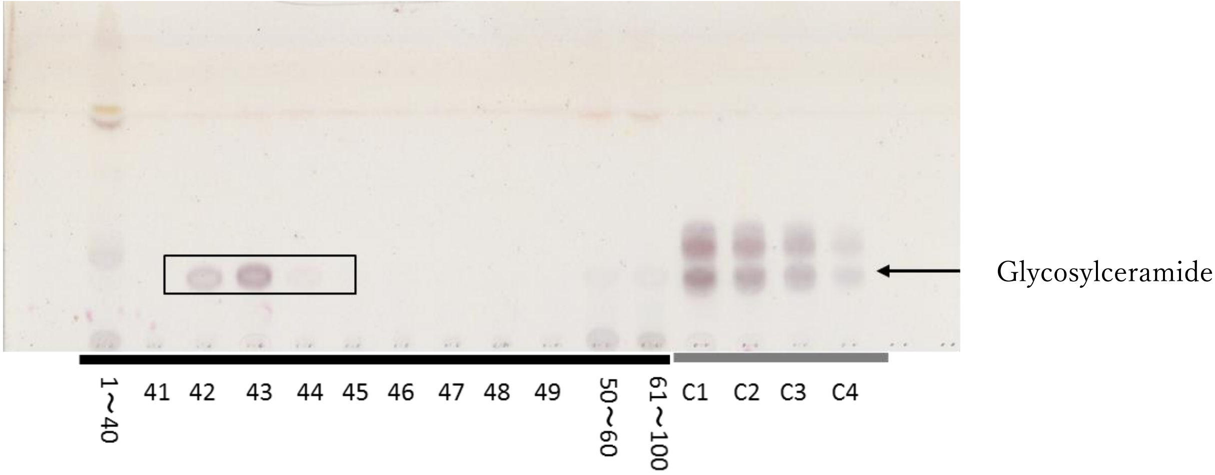

Fig. S1. Purification of glycosylceramide from mycelia of *A. oryzae*.
Total lipids were extracted from a 500 ml culture of *A. oryzae*, and purified using silicagel column chromatography and HPLC. Detailed methods are described in the text. Numbers indicate the fraction numbers. C1–C4 indicates standard cerebroside obtained from Matreya Inc. (C1: 23.1 μg, C2: 11.6 μg, C3: 5.8 μg, C4: 2.9 μg).
